# Clonidine as an adjuvant to bupivacaine in spinal anaesthesia for infra-umbilical surgeries in children: a prospective randomized, double-blind trial

**DOI:** 10.4314/gmj.v55i2.2

**Published:** 2021-06

**Authors:** Geeta Kamal, Raman Piplani, Anju Gupta, Nidhi Sehgal

**Affiliations:** 1 Department of Anaesthesia, Chacha Nehru Bal Chikitsalya, Geeta colony, Delhi – 110031, India; 2 Department of Anesthesiology, Pain Medicine and Critical Care, AIIMS, Delhi, India

**Keywords:** anaesthesia, adjuvant, bupivacaine, clonidine, child, spinal

## Abstract

**Objective:**

Spinal anaesthesia (SA) is a well-established technique for infra-umbilical surgeries but is underutilized in children. One important reason is the limited duration of action. Clonidine is a useful adjuvant in this regard but has not been studied in a dose of 1.5 µg/kg.

**Design:**

a prospective randomized study

**Setting:**

A single centre study conducted at a Super speciality paediatric tertiary care centre.

**Participants:**

Sixty children (5-12yrs) scheduled for lower abdominal surgery with duration <90min were included.

**Interventions:**

The participants were randomized into two groups to receive 0.4mg/kg of 0.5% hyperbaric bupivacaine with clonidine 1.5µg/kg (Group-I, n=30) or 0.4mg/kg of 0.5% hyperbaric bupivacaine with saline (Group-II, n=30) in the subarachnoid block.

**Main outcome measures:**

The sensory and motor block duration, time to two-segment regression, time to first rescue analgesic, and the number of rescue doses required were recorded.

**Results:**

Mean duration of sensory blockade (147.5±7.28 vs 310.33±10.17min; P<0.001) and motor blockade (132.5±10.06 vs 283.33±11.77min; P < 0.001) and duration of analgesia (172±9.61 vs 364.50±28.75min; P < 0.001) were significantly prolonged in the clonidine group. In the control group, most patients needed three analgesic doses over 24hr while in the clonidine group, the majority needed two doses. Adverse effects were infrequent in both groups.

**Conclusions:**

Clonidine as an adjuvant to 0.5% hyperbaric bupivacaine significantly prolonged the duration of analgesia with improved quality of anaesthesia while maintaining safety. We recommend the routine use of clonidine 1.5 µg/kg dose as an adjuvant to 0.5% bupivacaine in paediatric SA.

**Funding:**

None

## Introduction

The history of paediatric spinal anaesthesia (SA) dates back to 1898 when August Bier injected cocaine intrathecally in an 11-year-old child for surgery of thigh tumour and paved the way for this valuable technique in the field of paediatric anaesthesia.^1^ Ever since, it gained considerable interest and significance. Still, even after a substantial safety record of over a century, the role of spinal anaesthesia (SA) as a primary anaesthetic technique in children remains an anathema. It is confined to only a few specialized paediatric centres globally. The sole use of SA has been considered as a standard of care for sick ex-premature neonates and infants who are <60 weeks post-conceptional age to reduce the incidence of postoperative apnea when compared to general anaesthesia (GA).^2^,^3^

However, there is ample literature to suggest its safety and efficacy for suitable procedures in older children as well.^4^,^5^

SA in children shares the list of advantages observed in adults with an added advantage of minimal cardio-respiratory disturbance with lesser incidence of complications like Post-Dural Puncture Headache (PDPH).^2^ SA is a well-known technique for lower abdominal, urogenital, and orthopaedic surgeries. It is easy to perform, rapid in onset with effective sensory and motor block. The main deterrent to its application in children is its shorter duration due to the highly vascular pia mater.

High cardiac output leads to rapid re-absorption of local anaesthetic (LA) agents.^2^ This fact is reiterated by the 30% prolongation of the block by adding epinephrine to LA, unlike in adults.^6^

Therefore, a feasible solution to this would be the use of adjuvants to prolong local anaesthetic action. Though the adult literature is replete with clinical trials accomplished on evaluating a variety of adjuvants for SA, paediatric data largely remains deficient. An important consideration in children is the potential of neuraxial administered drugs to increase apoptosis in developing the spinal cord and inflict specific toxicity patterns by altering neural activity in the cord.^7^ It has been demonstrated that clonidine is safer in this regard and should be preferred as an adjuvant in children.^8^ Clonidine is a selective alpha-2 adrenergic receptor agonist that allows lower LA doses to be used with safety and prolongs postoperative analgesia. Clonidine has been used in paediatric SA at one µg/kg and two µg/kg.^9^,^10^,^11^,^12^,^13^ At a lower dose, clonidine may not achieve its optimal efficacy though associated with reasonable safety.

Similarly, at higher doses, 2 µg/kg and above, clonidine may show good efficacy at the cost of increased adverse effects. The efficacy and safety of clonidine in the dose of 1.5 µg/kg have not been investigated in children. The study was primarily aimed at finding the duration of postoperative analgesia obtained. We hypothesized that the addition of clonidine as an adjuvant to hyperbaric bupivacaine in SA would prolong the duration of analgesia without any significant adverse effects.

## Methods

This prospective randomized controlled study was conducted in a tertiary paediatric super speciality hospital based in North India. Approval from the Institutional ethics committee (Maulana Azad Medical College and associated hospital institutional ethics committee, approval no. F.1/IEC/MAMC/2017/No 123) and written informed consent was duly obtained from the parents/guardian of the children.

**Inclusion criteria:** The study enrolled 60 children of ASA physical status I and II, aged 5-12 years belonging to either sex, scheduled for lower abdominal surgery with duration not exceeding 90 minutes (inguinal hernia, orchidopexy, posterior, urethroplasty, urethral valve excision etc.).

**Exclusion criteria:** Patients with bleeding diathesis, drug allergy, contraindications to regional anaesthesia, nerve injury, major systemic illness, psychiatric disease and patients on anticoagulants and sedative drugs were excluded.

**Randomization and blinding:** Patients were randomized to one of the two groups using computergenerated random number sequence maintained in sequentially numbered sealed opaque envelopes. The study drug was prepared by an independent anaesthetist in a separate drug preparation area as per group allocation while following all aseptic measures and handed over to the person administering the intrathecal drug. This person did not participate in the study further. Hence, the patient, the person administering the intrathecal drug and the outcome assessors were all blind to the group allocation. The two allocated groups received the following drugs as per randomization:

Group I: N=30 patients to receive 0.4 mg/kg of 0.5% hyperbaric bupivacaine (Anawin Heavy, Neon Labs Ltd., Mumbai, Maharashtra, India) and preservative-free clonidine 1.5 µg/kg (150 µg/ml 1ml ampule; Neon Labs Ltd., Mumbai, Maharashtra, India)
Group II: N=30 patients to receive 0.4 mg/kg of 0.5% hyperbaric bupivacaine (Neon Labs Ltd., Mumbai, Maharashtra, India) and an identical volume of preservative-free saline

All patients were confirmed for minimum fasting of six hours for solid food and two hours for clear fluids prior to surgery. It was also ensured that the children empty their bladder before the operation. Oral Midazolam syrup (2mg/ml; Neon Labs Ltd., Mumbai, Maharashtra, India) premedication of 0.5 mg/kg was given 30 minutes before surgery. Standard monitoring for Heart rate (HR), Noninvasive blood pressure (NIBP) and Oxygen saturation (SpO_2_) were established. The temperature of the operation room was kept at a comfortable level. The baseline parameters, including sedation score, were recorded (0: awake and agitated; 1: Awake and comfortable; 2: Asleep but arousable; 3: Asleep with sluggish response to a verbal command or touch and 4: No response to verbal command or touch).

A 20 or 22 G intravenous (IV) cannula as appropriate for age was secured, and Ringer's lactate solution started. A neutral position of the operation table was ensured. The patients were sedated with oxygen (O_2_) and Nitrous oxide (N_2_O) (50:50) mixture and sevoflurane (3-4%) with a mask connected to Jackson Rees (JR) modification of Ayer's T piece or Bain's circuit as deemed appropriate. The patients were positioned in the left lateral decubitus position. Before attempting spinal puncture, 1mg/kg IV propofol bolus (10mg/ml formulation, Troikaa pharmaceuticals ltd., Ahmedabad, Gujrat, India) was administered to facilitate the procedure. Experienced paediatric anaesthesiologists performed all the blocks.

After adequate aseptic precautions and dermal infiltration of lidocaine 1% (Neon Labs Ltd., Mumbai, Maharashtra, India), a lumbar puncture was performed at L3-L4 intervertebral space with a 90 mm, 25-Guage Quincke Babcock spinal needle (Becton Dickinson S.A, Madrid, Spain). After ensuring a free flow of CSF, patients were given the prescribed combination of study drugs intrathecally slowly over approximately 20 seconds.

The end of the injection was taken as time ‘0’. Immediately after the block, patients were placed in the supine position. Sevoflurane and N_2_O were discontinued. Patients were given oxygen by facemask when breathing adequately. The adequacy of ventilation was monitored using the end-tidal carbon dioxide monitoring. The procedure of raising limbs for electrocautery pad placement altogether avoided for 5 minutes. The surgeons were communicated before block placement to be washed and scrubbed up to save time.

Henceforth, the sensory block was assessed with pinprick using a blunted hypodermic needle (caudal to cephalic), while the motor block was assessed using a modified Bromage scale. They were recorded bilaterally at 30 seconds for the first 3 minutes and then every 2 minutes for 10 minutes. The adequacy of SA was determined by the absence of facial grimacing on gentle pinprick and the presence of profound motor block at the level of hips. Patients were given GA if the effect was found inadequate.

The vital parameters and sedation score were periodically monitored and recorded at the 5-minute interval for the first 30 minutes, at the 15-minute interval for the first 60 minutes and 30-minute interval for the next 2 hours. After that, it was done at hourly intervals for 6 hours and again at 12 hours. Clinically relevant bradycardia was defined as an HR of less than 60/min and treated with iv atropine 20 µg/kg. Clinically relevant hypotension was defined as a decrease of 20% in mean arterial pressure from the baseline and appropriately treated with fluids and IV mephentermine 3mg if required.

Maintenance fluids were duly given as per Holliday and Segar formula. Episodes of respiratory depression (SpO_2_<94%) was treated with oxygen supplementation, and intraoperative awakening and agitation were addressed with IV midazolam 0.05–0.1 mg/kg, respectively. Injection Ondansetron 0.1 mg/kg was given iv as antiemetic prophylaxis towards the end of surgery. All preparations were made to treat vomiting, respiratory depression, urinary retention, and high spinal, should they occur. In case of failure, GA was kept as a backup.

Postoperatively, the block was assessed at specified time intervals for the study. During the regression phase, time to two-segment regression and time for regression of sensory block to L2 (Duration of the sensory block) were recorded. The time to full recovery of motor block (Duration of the motor block) was also recorded. The pain was measured in severity using a 10 cm visual analogue scale (VAS) or FACES scale at specified intervals for the next 24 hours by a resident doctor who was unaware of the group. Effective analgesia was defined as the time duration from onset of the sensory blockade to the first demand of rescue analgesic at VAS/FACES score ≥ 4.

Injection paracetamol 15 mg/kg IV was given as rescue analgesia. If pain score failure decreased below 4, tramadol 1mg/kg was administered IV. The time for first rescue analgesia and the number of rescue doses required were also recorded. Sedation was assessed using the Ramsay Sedation Scale at specified periods. The elapsed time between intrathecal injection and spontaneous urination was recorded. Adverse effects such as hypotension, bradycardia, postoperative nausea and vomiting (PONV), respiratory depression, urinary retention and headache were recorded postoperatively.

### Sample size calculation

The duration of analgesia was our primary outcome measure of interest. A previous study by Kaabachi et al.^10^ documented the mean (SD) for the duration of analgesia to be 330 (138) minutes in children undergoing surgery under spinal anaesthesia. Assuming that the addition of clonidine will improve the duration of analgesia by 30%, with the permitted alpha error of 0.05 and beta error of 0.2 and the study power of 80 %, a minimum sample size of 30 patients were required per group. Hence, we decided to recruit a total of 60 patients.

### Statistical analysis

The quantitative variables are expressed in terms of mean ± SD and compared between groups using unpaired t-test. Qualitative variables are expressed as frequencies/percentages and compared between groups using Fisher's Exact/Chi-square test. A p-value < 0.05 was considered statistically significant. The data were tabulated using MS Excel package while statistical analysis was performed on SPSS version 16.0 software.

## Results

**Figure 1:**
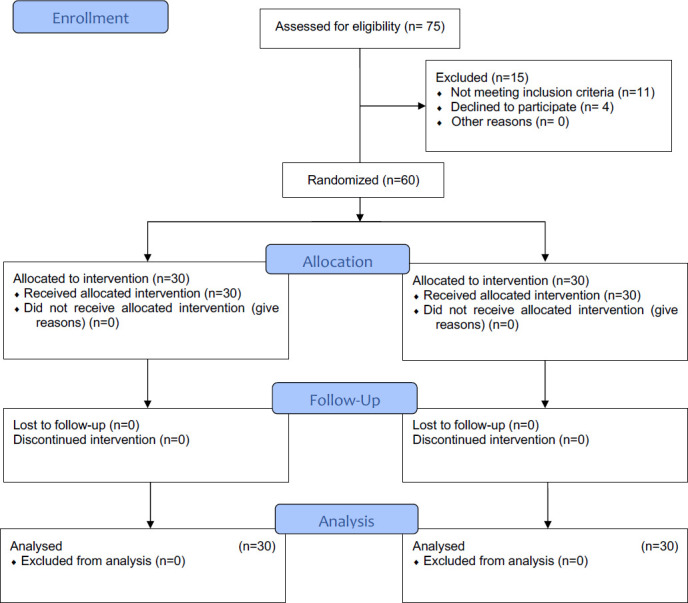
CONSORT flow diagram

Seventy-five children were assessed for eligibility and we included sixty patients who fulfilled the study's inclusion criteria (CONSORT diagram, Figure 1). None of the patients required conversion to GA during the study. The patient characteristics in terms of age, gender and weight were comparable among the patients in both groups (Table 1)

**Table 1:** Patient characteristics (mean and Standard deviation (SD))

Patient characteristics	Control (N=30)	Clonidine (N=30)	p-value
**Age (years)**	8.33 ± 2.12	7.87 ± 1.84	0.183
**Gender (M/F)**	22/8	19/11	0.203
**Weight (Kg) (mean± SD)**	23.10 ± 4.90	22.77 ± 6.18	0.409
**Duration of surgery (min) (Mean± SD)**	62.07 ±17.55	59.77 ± 20.72	0.324

The block characteristics comparing both groups depicted significantly prolonged duration of sensory and motor block and effective analgesia in Group I. The duration of sensory and motor block respectively was 310.33±10.17 and 283.33±11.77 minutes in Group I and 147.50±7.28 and 132.50±10.06 minutes in Group II (P ≤ 0.001). The duration of effective analgesia was 364.50±28.75 and 172.00±9.61 minutes (P ≤ 0.001) in Group I and Group II, respectively. (Table 2)

**Table 2:** Block characteristics, rescue analgesia in the patients

Parameters	Control (N=30)	Clonidine (N=30)	p-value
**Sensory block in min (mean± SD)**	147.50 ± 7.28	310.33 ± 10.17	<0.001
**Motor block in min (mean± SD)**	132.50 ± 10.06	283.33 ± 11.77	<0.001
**Effective analgesia in min (mean± SD)**	172.00 ± 9.61	364.50 ± 28.75	<0.001
**Intraoperative awakening (N)**	12	6	0.045
**No. Of rescue analgesic doses(N)**			
**2**	3	18	<0.001
**3**	21	11	0.005
**4**	6	1	0.002
**No. Of analgesic doses (N)**	3.10 ± 0.55	2.43 ± 0.57	<0.001
**Time to rescue analgesics in hours (mean± SD)**	2.87 ± 0.16	6.08 ± 0.48	<0.001

On comparison, Group I depicted only six patients (20%) who awakened intraoperatively against twelve patients (40%) in Group II. This was also statistically significant (P = 0.045)

The analgesic requirement was significantly reduced in Group I as compared to Group II. On comparison, three patients (10%) in Group II while eighteen patients (60%) in Group I required two doses. Twenty-one patients (70%) in Group II as against eleven patients (36.67%) in Group I required three doses while six patients (20%) in Group II and only one patient (3.33%) in Group I required four doses of rescue analgesia (P <0.001) (Table 2).

In Group II, 26 patients (86.67%) required rescue analgesics between 2-3 hours of subarachnoid block whereas none required in Group I (P <0.001). In Group I, 16 patients (53.33%) required rescue analgesics between 5-6 hours while 14 patients (46.67%) required doses between 6-7 hours. During these hours, the comparison of both groups was statistically significant. On comparison, the difference between the mean number of rescue analgesic doses (P <0.001) and the mean time of requirement of analgesic doses (P <0.001) were also statistically significant(Table 2).

Bradycardia was the only complication which depicted statistical significance amongst both the groups. Three patients (10%) in Group I developed bradycardia as against none (0%) in Group II (P=0.038). One patient (3.33%) developed hypotension in both the group (P=0.500). Hypotension and vomiting were observed in one patient (3.33%) each in both the groups (P=0.500). Five patients (16.67%) developed post-dural puncture headache (PDPH) in Group I against two patients (6.67%) in Group II (P=0.114). Overall, seven patients (11.67%) developed a headache in the study. (Table 3)

**Table 3:** Complications (Intra and postoperative)

Complications	Control (N=30)	Clonidine (N=30)	P value
**Bradycardia**	0	3	0.038
**Hypotension**	1	1	0.500
**Nausea/Vomiting**	1	1	0.500
**Respiratory depression**	0	0	-
**Urinary retention**	0	0	-
**PDPH**	2	5	0.114
**High/total spinal**	0	0	

## Discussion

The present study demonstrated that the use of clonidine in a dose of 1.5 µg/kg when added to hyperbaric bupivacaine intrathecally, resulted in a statistically significant increase in the duration of sensory and motor block as well as effective postoperative analgesia when compared to bupivacaine alone. The subarachnoid block is a well-established technique for infra-umbilical surgeries. SA is technically simple, produces rapid onset profound and uniformly distributed analgesia with effective neuromuscular blockade and minimal risk of toxicity. It avoids GA, airway manipulations, reduces the stress response and enhances recovery.

SA has been recommended as an alternative anaesthetic in the paediatric population ranging from preterm infants to adolescents. Krane et el.^3^ had evaluated infants undergoing hernia repair receiving SA or GA and found no alteration in the respiratory patterns in the spinal group, whereas GA was associated with decreased oxygen saturation and heart rate. It reduces the incidence of morbidity that follows general anaesthesia in preterm and infant population.

Bupivacaine is the most common local anaesthetic for spinal anaesthesia in children, but hyperbaric bupivacaine produces short-lasting spinal anaesthesia in children which may be insufficient for the planned procedure.^2^ Clonidine is an alpha-2 adrenergic receptor agonist, has remarkable synergistic effects as an additive and increases the efficacy of sensory and motor block in a dose-dependent manner. The analgesic effect following intrathecal administration is mediated spinally through activation of postsynaptic alpha-2 receptors in substantia gelatinosa of the spinal cord. Intrathecal clonidine achieves a higher drug concentration in the vicinity of alpha 2 adrenoceptor in the spinal cord. It acts by blocking the conduction of C and A-delta fibres, particularly by motor neuron hyperpolarization and hence intensify conduction blockade by LAs. It also reduces calcium conduction in cells, thus inhibiting neurotransmitter release. Doses 1 µg/kg and 2 µg/kg have been studied in various age groups.

As an adjunct to spinal anaesthesia clonidine 1µg/kg used in previous studies also, showed that clonidine provides a significant improvement in spinal anaesthesia quality, increases the duration of analgesia and reduces the need of postoperative analgesic requirements without undesirable side effects similar to our study.^9^,^10^,^11^,^12^ Duration of postoperative analgesia reported in studies on similar age group children coincided with our study (365±84 min and 360 ± 31min as compared to 364.50±28.75min in our study).^11^,^12^

A study using 2 µg/kg clonidine in spinal anaesthesia concluded that the addition of clonidine to bupivacaine yielded long-lasting pain relief (490 +/- 35 minutes).^13^ However, the incidence of hemodynamic effects was unacceptably high (30% bradycardia and 52% hypotension).

The age group of 5-12years is vulnerable to perioperative anxiety and less likely to cooperate for the lumbar puncture and the surgery.

We used oral midazolam 0.5 mg/kg as a safe, effective, and acceptable premedication.^2^ This ensured cooperation, greater comfort, and motor control during the procedure. Inadvertent patient movement can also lead to harm to neural structures while the needle tip is in subarachnoid space and excessive spread of the block. We used light inhalation anaesthesia and a small dose of propofol to ensure the absence of movement during puncture. A similar technique was used by Imbelloni et al. who found it quite suitable.^5^ The need for sedation during lumbar puncture may be decreased with a eutectic mixture of local anaesthetic (EMLA) application for dermal analgesia. Although it is a better option, it could not be made available in our setup and we had to use intradermal lignocaine infiltration.

As compared to plain bupivacaine group, there was a lesser need for intraoperative sedation and fewer incidents of intraoperative awakening in the clonidine group. (P<0.05) The systemic absorption of clonidine may have resulted in sedation due to alpha-2 receptor agonism at locus coeruleus. In the postoperative period also, the patients in the clonidine group were calmer and more comfortable as was evident by the higher sedation score. However, at no time did the sedation score exceed >2 and none developed respiratory depression. Similar results have been reported in the study by Cao JP et al. on 6-8 years children undergoing orthopaedic surgery.^14^ They demonstrated better postoperative analgesia, improved sedation and reduced intraoperative requirement of propofol with intrathecal clonidine.

The position for lumbar puncture depends on the age and the need for sedative premedication. The lateral position may be easier for older children who generally require sedation. The raising of limbs for any purpose after the block placement and Trendelenburg position must be avoided as it may lead to high spinal after placement of the block.^15^ Hence, we adhered to a no-patient movement policy for at least five minutes after a successful puncture.

Dohi S et al. concluded that children less than five years of age showed little or no change in blood pressure and heart rate following sympathectomy while older children behave more like adults.^16^ We excluded children <5 years in our study to maintain a uniform physiological response of sympathetic blockade in the study population.

In this study, we used hyperbaric bupivacaine 0.5% at a fixed dose of 0.4 mg/kg which is the recommended dose for 5-15kg children.^2^ The total volume of the drug combination was kept identical in both the groups.

The main limitation of spinal anaesthesia is a variable and relatively short duration of the block with a single shot of LA.^2^ In children, SA is commonly accepted for procedures with a duration of 60-75 minutes, up to a maximum duration of 90 minutes.

The upper limit of duration in our study was 90 minutes and procedures expected to exceed this duration were excluded.

One of the side effects of clonidine is bradycardia. The stimulation of alpha-2 receptors in the medullary vasomotor centre after systemic absorption is responsible for a decrease in sympathetic outflow and cardiovascular side effects. There were 3 episodes of bradycardia noted in Group I as against none in Group II. The events responded immediately to atropine and did not result in hemodynamic instability.

Only one episode of hypotension (3.33%) was observed in each group. The patient in Group II developed hypotension immediately after a difficult lumbar puncture which may be explained by the combined effect of inhalational agents used for a prolonged period during puncture and SA induced sympathectomy. Similarly, Group I patient who developed hypotension had a sudden episode of excessive bleeding. Incidence of hypotension did not show statistical significance and the cause of hypotension cannot be fully attributed to the use of clonidine or subarachnoid blockade itself. Both the patients responded to iv fluids alone. Previous studies (Rochette et al.^13^ and Kabaachi O et al.^10^ where a higher dose of clonidine was used (2 µg/kg) as an additive found very frequent and severe episodes of bradycardia and hypotension than studies by Rochette et al.,^9^ Kabaachi O et al.,^10^ Cham et al.^12^ using lower doses (1 µg/kg) clonidine doses though it yielded long-lasting pain relief.^9^,^10^,^12^,^13^, Rochette et al. in their dose-finding study on intrathecal clonidine in neonates found a higher incidence of respiratory depression which needed treatment with caffeine in neonates receiving 2 µg/kg dose as compared to 1 µg/kg.^9^

Incidence of headache was comparable in the two groups and the overall incidence was 11.67%. Our results are similar to those of Wee et al. who reported an incidence of 11.8% in children 10-12 years of age.^17^ We used 25 G Quincke needle due to non-availability of pencil-point needles. A study by Kokki et al. comprising children aged 8 months to 15 years comparing the incidence of headache with a pencil point and cutting point needle showed no difference between the two.^18^ All patients were adequately hydrated and advised the supine position until discharge. No other adverse effects like desaturation, apnoea or urinary retention were seen.

There are certain limitations to our study. We have compared only a single dose of clonidine based on literature search and the optimal dose may be different from what we have studied. However, comparison with the results of previously available literature suggests that the current dose may be the optimal dose. The study was powered for the primary outcome i.e., duration of analgesia. However, it may be underpowered to detect a significant difference in other outcomes like bradycardia.

## Conclusion

Clonidine as an adjuvant to 0.5% hyperbaric bupivacaine significantly prolonged the duration of analgesia and also improved the quality of anaesthesia while maintaining safety. We recommend the routine use of clonidine 1.5 µg/kg dose as an adjuvant to 0.5% bupivacaine in paediatric SA.
